# Cultivating Bacteria's Taste for Toxic Waste

**DOI:** 10.1371/journal.pbio.0040282

**Published:** 2006-08-08

**Authors:** Liza Gross

More than 50 years after developing the nuclear bomb, the US Department of Energy is still grappling with the toxic consequences of nuclear weapons production. By the agency's own reckoning, more than 2,500 billion liters of groundwater and 200 million cubic meters in 30 states and territories are contaminated with uranium. Based on increasing evidence that microorganisms can transform contaminants as a natural by-product of cellular metabolism, the agency launched an ambitious program in 1995 aimed at harnessing natural biogeochemical processes to clean up radioactive sites.

Dissimilatory metal-reducing bacteria (DMRB) can convert soluble radioactive uranium into an insoluble, or solid, form called uraninite. The soluble form moves through groundwater with relative ease; the insoluble form can stick to soil particles and is far less mobile, significantly reducing the probability that uranium will reach surface water or aquifers used for domestic water supply. But, before the bioremediation potential of DMRBs can be exploited, scientists must work out many details of uranium reduction by these microbes and determine the best ways to increase their fancy for toxic heavy metals. In a new study, Matthew Marshall, James Fredrickson, and colleagues examine the mechanisms of uranium reduction in a microbe that's widely used in environmental research called *Shewanella oneidensis* strain MR-1. Using diverse genetic and microscopy techniques, they show that two polyheme *c*-type cytochromes, MtrC and OmcA—proteins known to be involved in iron and manganese reduction—are also required for the reduction of uranium to insoluble uraninite.

*S. oneidensis* has a remarkably flexible respiratory network. When oxygen is available, the microbe obtains energy by oxidizing organic compounds to carbon dioxide, using oxygen as the electron receptor. When oxygen is scarce—in heavy-metal-laden sediments, for example—the microbe oxidizes organic compounds to carbon dioxide by using metals such as iron or uranium as electron acceptors. These “redox” reactions transfer electrons from one molecule (which is oxidized) to another (the reduced “terminal electron acceptor”) with the help of cytochromes as part of the terminal reductase complex.

Because uraninite accumulates on the surface of the microbe's outer membrane, Marshall et al. reasoned that outer membrane cytochromes (OMCs) might contribute to the formation of uraninite nanoparticles. To investigate the role of OMCs in uranium reduction, they first studied a *S. oneidensis* mutant lacking the ability to produce functional *c*-type cytochromes (the microbe has 42 genes predicted to make *c*-cytochromes). This mutant failed to reduce uranium during a 48-hour period, whereas the nonmutant microbe reduced equivalent amounts of uranium totally in 24 hours. The researchers also created four OMC deletion mutants; those lacking either MtrC or OmcA, or both OMCs, had significantly slower uranium reduction rates. The fact that MtrC and OmcA affect uranium reduction rates suggests both proteins are important, even though in vitro experiments showed that only MtrC can function as a terminal reductase to transfer electrons to uranium. Deleting another OMC, *mtrF*, had little effect on reduction rates, which was not surprising, since this gene has never been linked to electron transfer to metals.

The researchers next used microscopic analysis on nonmutant and mutant microbes to determine what effect the OMC deletions had on the cellular location of uraninite particles. In nonmutants, uraninite is found localized both extracellularly and between the cell's inner and outer membranes (the periplasm). A large proportion of the extracellular uraninite was densely packed in association with a complex called extracellular polymeric substance (EPS). In contrast, OMC mutants typically accumulated more uraninite in their periplasm rather than consorting with EPS outside the cell. Mutants lacking just MtrC or both MtrC and OmcA also revealed the most significant differences in the abundance, distribution, and density of uraninite–EPS complexes.

X-ray fluorescence microscopy revealed that elemental iron was closely associated with the uraninite–EPS complex, suggesting the presence of an iron-containing protein—such as heme-containing OMC(s)—was located with the complex. Using a combination of high-resolution microscopy and specific antibodies to examine OMC localization relative to the uraninite complexes, the researchers found both OmcA and MtrC time and again co-localized together and with the uraninite–EPS complexes.

Though more experiments are needed to identify the role of other agents involved in uranium reduction, including that of periplasmic cytochromes, it is clear that MtrC and OmcA play a major part in uranium transformation to insoluble nanoparticles in *S. oneidensis*. Based on these results, the researchers believe that uraninite associations with complex biopolymers such as EPS in the environment could slow the re-oxidation of uranium and prevent nanoparticulate uraninite from dispersing in groundwater. Noting the plethora of predicted *c*-type cytochromes in the microbe's genome, the researchers suspect that many more can transfer electrons to uranium. The nature of these cytochrome–uraninite associations may well determine whether bioreduction of uranium-contaminated soil can protect water resources over the long-term.

## 

**Figure:**
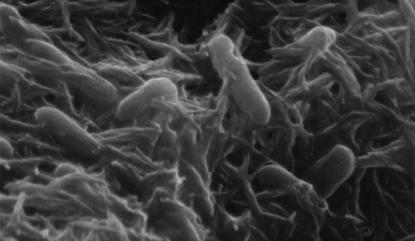
The bacterium *Shewanella oneidensis* strain MR-1 (above) may offer a biological solution for remediating US sites contaminated during the manufacture of nuclear weapons.

